# Effects of eight weeks of resistance training on the risk factors of metabolic syndrome in overweight /obese women - “A Pilot Study”

**DOI:** 10.1186/1758-5996-5-11

**Published:** 2013-02-28

**Authors:** Ramires Alsamir Tibana, James Navalta, Martim Bottaro, Denis Vieira, Vitor Tajra, Alessandro de Oliveira Silva, Darlan Lopes de Farias, Guilherme Borges Pereira, Jéssica Cardoso de Souza, Sandor Balsamo, Claudia Regina Cavaglieri, Jonato Prestes

**Affiliations:** 1 Graduate Program on Physical Education, Catholic University of Brasilia, Q.S. 07 Lote 01 – Bloco G, 71966-700, Brasilia, Brazil; 2 Department of Physical Education, Euro-American University Center (UNIEURO), Brasilia, Brazil; 3 Kinesiology and Nutrition Sciences, University of Nevada, Las Vegas, USA; 4 College of Physical Education, University of Brasilia, Brasilia, Brazil; 5 School of Physical Education, State University of Campinas, Campinas, Brazil; 6 Graduate Program in Medical Sciences of the University of Brasilia, School of Medicine – UnB, Brasilia, DF, Brazil

**Keywords:** Resistance training, Obesity, Overweight, Metabolic syndrome

## Abstract

**Background:**

The purpose of the present study was to examine the effects of eight weeks of resistance training (RT) on anthropometric, cardiovascular and biochemical risk factors of metabolic syndrome (MetS), and neuromuscular variables on overweight/obese women.

**Methods:**

Fourteen middle-aged (33.9 ± 8.6 years) overweight/obese women (body mass index - BMI 29.6 ± 4.1 kg/m^2^) underwent 24 sessions (3 times/week) of a whole body RT program with 3 sets of 8–12 repetitions maximum (RM). The following variables were evaluated: maximum strength on chest press and frontal lat pull-down; isometric hand-grip strength; biceps brachii (BB) and rectus femoris (RF) muscle thickness, body mass; BMI; body adiposity index (BAI); waist, hip and neck circumferences; visceral fat volume; blood glucose; glycated hemoglobin (HbA1c); insulin; HDL-C and triglycerides.

**Results:**

There was an increase of chest press (from 52.9 ± 9.7 to 59.8 ± 7.7 kg; P = 0.02) and front lat pull-down (from 51.5 ± 7.5 to 57.6 ± 9.2 kg; P = 0.01) muscle strength, isometric handgrip (P = 0.02) and RF muscle thickness (from 42.2 ± 8.5 to 45.1 ± 7.3 mm; P = 0.02) after the 8 week RT program. There were no statistically significant alterations on plasma glucose, HbA1c, insulin, triglycerides, HDL-C, anthropometric indexes and BB muscle thickness (p > 0.05).

**Conclusions:**

A RT program without caloric restriction promotes an increase on muscle thickness and strength, with no effects on risk factors of MetS in overweight/obese women.

## Introduction

The prevalence of Metabolic Syndrome (MetS) has been increasing worldwide, in parallel with the increasing prevalence of obesity. MetS is characterized by the grouping of several cardiovascular risk factors such as: abdominal obesity, hypertension, insulin resistance, glucose intolerance/type 2 diabetes, and dyslipidemia [1]. Furthermore, various epidemiologic studies have shown strong associations between these risk factors and the development of other chronic diseases problems such as gastrointestinal cancer [2], diabetes [3], cardiovascular disease (CVD) [4], or even premature mortality [5,6]. Thus, the development of strategies to prevent and treat MetS, overweight and obesity are of great importance.

Sedentary behavior which includes activities such as lying down, sitting, watching television, using the computer, and other forms of screen based entertainment are positively associated with an increased risk of type 2 diabetes [7,8], cancer [9], MetS [10], and all-cause and CVD mortality [7,8]. Therefore, lifestyle modifications [11] and exercise have been consistently recommended for the treatment and prevention of hypertension and metabolic diseases. Aerobic exercises such as walking and bicycling have been widely used to prevent the development of metabolic and cardiovascular risk factors, MetS, type 2 diabetes, CVD, and premature mortality [12].

Nevertheless, the inclusion of resistance training (RT) as an integral part of an exercise program which also includes aerobic or combined exercise has been endorsed by the American Heart Association [13], the American College of Sports Medicine [14] and the American Diabetes Association [15]. Recent studies have suggested that lower levels of muscular strength are associated with the prevalence of MetS [16,17], obesity [18], hypertension [19] and premature mortality [20]. Moreover, RT for persons with MetS induces no additional systemic elevation of pro-inflammatory cytokines [21] and is effective in reducing clinical and 24 h blood pressure in middle-aged overweight/obese women [22]. However, Stensvold *et al*., [23] found minimal effects of RT on risk factors of MetS. The conflicting data reinforce the necessity of more studies investigating the effects of RT on the risk factors of MetS.

The purpose of the present study was to examine the effects of eight weeks of RT on anthropometric, cardiovascular and biochemical risk factors of MetS, and neuromuscular variables in overweight/obese women. The initial hypothesis was that chronic RT without dietary restriction would improve neuromuscular variables and decrease some risk factors of MetS, such as blood pressure and waist circumference.

## Methods

### Subjects

**Figure 1:**
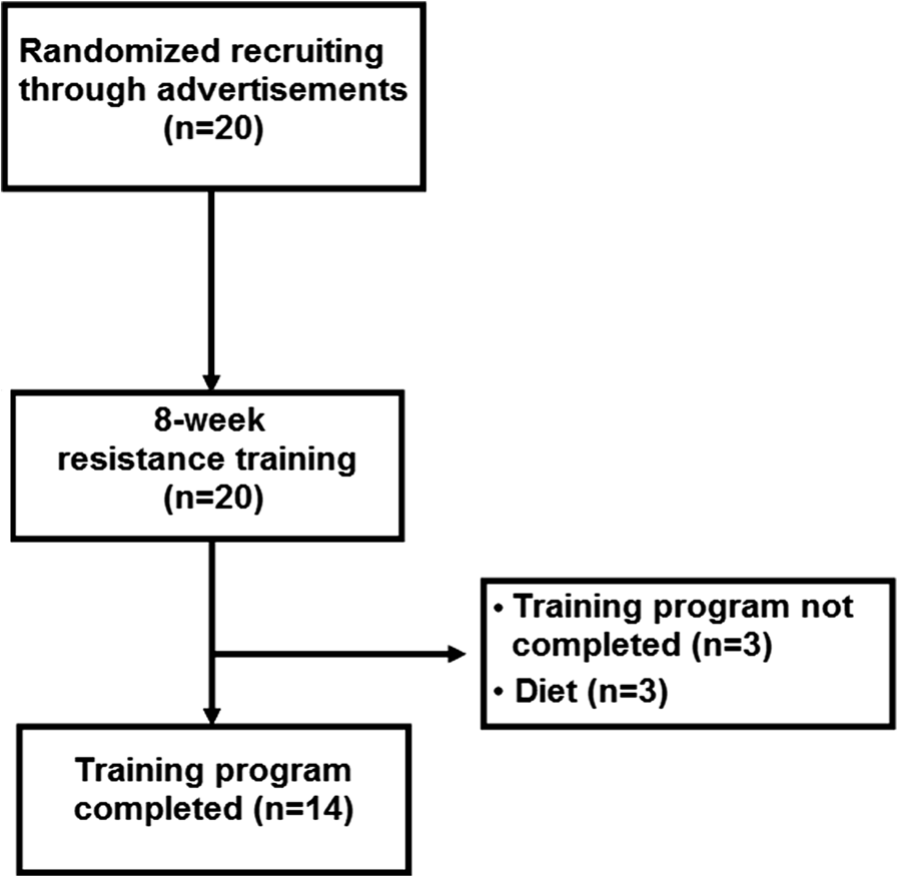
**Scheme of the study design.** Diagram of the selection process of the volunteers.

Initially, 20 women from the local community volunteered to participate from posters and lectures about the study. However, only 14 completed the study, with three volunteers excluded due to caloric restriction and three excluded from the statistical analysis because they missed more than 25% of the training sessions (Figure 1). Individuals completed a thorough physical examination, including a medical history, resting and exercise electrocardiogram [24], blood pressure assessment, anthropometric, and orthopaedic evaluation prior to participation in the experimental protocols. As inclusion criteria, the only participants included were those aged between 18–40 y, classified as overweight (N = 9) or obese (N = 5) by BMI measurement according to the World Health Organization (WHO): overweight BMI = 25.0–29.9 kg · m^2^ and obese > 30.0 kg · m^2^, and those without consistent RT for the past six months before the study period. Women with physical disabilities, under caloric restriction, diagnosis of diabetes, cardiovascular diseases, hypertension (systolic blood pressure > 140 mmHg and diastolic blood pressure > 90 mmHg) [25], musculoskeletal disease, recent use of medication and smoking or drug/alcohol abuse were excluded from the trial. Sedentary state was defined by the International physical activity questionnaire (IPAQ). All participants signed an informed consent document and the study was approved by the Catholic University of Brasilia Research Ethics Committee for Human Use (protocol #279/2010).

### Study design

The present study was designed to investigate the effects of eight weeks of RT on muscle thickness and strength, anthropometric, biochemical and cardiovascular risk factors of MetS in overweight/obese women. All testing and training sessions were conducted between 08:00–09:00 pm. Subjects were advised to maintain their normal daily eating habits throughout the study (this was guaranteed by a dietary recall follow-up).

Prior to physical evaluation, participants reported to the laboratory between 08:00–10:00 am following an overnight fast, for blood sampling from the antecubital vein for subsequent analysis of biochemical variables. Anthropometric variables, isometric handgrip strength, muscle thickness by ultrasound and thorough physical examination were determined. Volunteers completed two weeks of familiarization prior to testing (3 sessions/week, with one exercise for each main muscle group which were the same exercises used during RT), where they were advised regarding the execution of proper technique. After the familiarization period, one-repetition maximum (1-RM) test and re-test were performed on the chest press and front lat pull-down on two nonconsecutive days with 48–72 hours between tests. The RT protocol began three days after 1-RM testing and was performed on three non-consecutive days of the week, comprising three sets of 8–12 repetition maximum (RM) of twelve exercises, and 1-minute rest intervals between sets and exercises.

### Maximal strength testing

After 2 weeks of adaptation to the exercises and 3–5 days after the last training session, 1-RM tests were performed on 2 different days separated by a minimum of 48 h. All tests were performed with 10 min rest intervals between each exercise. The order was as follows: chest press and front lat pull-down (JOHNSON, USA). The protocol consisted of a light warm-up of 10 min of treadmill running followed by 8 repetitions at 50% of estimated 1-RM (according to the participants’ capacity verified in the 2 weeks of adaptation). After a 1-min rest, subjects performed 3 repetitions at 70% of the estimated 1-RM. Following 3 minutes of rest, participants completed 3–5 attempts interspersed with 3-to-5 min rest intervals, with progressively heavier weights (~5%) until the 1-RM was determined. The range of motion and exercise technique were standardized according to the descriptions of Brown and Weir [26].

### Isometric handgrip strength

Isometric handgrip strength was determined by a manual mechanical dynamometer (Takei, T.K.K Grip strength dynamometer 0–100 kg, Japan), according to the recommendations of Heyward [27]. Volunteers stood still with both arms extended and the forearm positioned in a neutral rotation. The handgrip width of the dynamometer was individually adjusted for each participant, according to hand size allowing the stem next to the body to be positioned on the second phalanges: index, medial and ring fingers. Three attempts were allowed interspersed with 1 min rest intervals. The best measure was used and relative isometric strength was determined as follows:

$$\mathrm{Relative}\;\mathrm{strength}=\mathrm{Absolute}\;\mathrm{strength}\;\left(\mathrm{kg}\right)/\mathrm{Body}\;\mathrm{mass}\;\left(\mathrm{kg}\right)$$

### Muscle thickness

Muscle thickness of the rectus femoris (RF) and biceps brachii (BB) were tested before and after the eight-week RT period. All tests were conducted at the same time of day, and participants were instructed to hydrate normally 24 h before the tests. Measures were taken 3–5 days after the last training session to prevent any residual effects (i.e. swelling) that could interfere with muscle thickness [28]. Participants were instructed to avoid any other type of exercise or intense activity. Muscle thickness was measured using B-Mode ultrasound (Philips-VMI, Ultra Vision Flip, model BF). A water-soluble transmission gel was applied to the measurement site and a 7.5-MHz ultrasound probe was placed perpendicular to the tissue interface while not depressing the skin. Muscle thickness of the use only RF and BB muscle from the dominant limb were measured according to the recommendations of Abe *et al*., [29]. Once the technician was satisfied with the quality of the image produced, the image on the monitor was frozen. With the image frozen, a cursor was enabled in order to measure muscle thickness, which was taken as the distance from the subcutaneous adipose tissue-muscle interface to muscle-bone interface [29]. A trained technician performed all the analysis.

### Total muscle mass

Total muscle mass was estimated according to the equation proposed by Lee *et al*., [30]:

$$\begin{array}{c} \mathrm{Skeletal}\;\mathrm{muscle}\;\mathrm{mass}\;\left(\mathrm{kg}\right){=\mathrm{Ht}}_{m}\left(0.244\times \mathrm{BM}\right)\\ \;+\left(7{.8\times \mathrm{Ht}}_{m}\right)+\left(6.6\times \mathrm{gender}\right)\\ \;–\left(0.098\times \mathrm{age}\right)+\left(\mathrm{ethnicity}–3.3\right) \end{array}$$

Where: Ht_m_, height (m); BM, body mass (kg); gender: male = 1, female = 0; ethnicity: Asian = 1.4, African-American = 1.2, White = 0.

### Anthropometric variables

Height and weight were measured for the calculation of the body mass index (BMI). All circumferences were obtained using non elastic tape, and measurements were obtained in triplicate and averaged to obtain the circumference score. Neck circumference was obtained with the subject sitting with the head in the Frankfort horizontal plane position. Briefly, a measuring tape was applied around the neck inferior to the laryngeal prominence and perpendicular to the long axis of the neck, while the minimal circumference was measured and recorded to the nearest 0.1 cm [31]. Waist circumference was measured at the midpoint between the lower rib margin and the (Yang et al., 2010). Body adiposity index (BAI) was determined by the following formula: (BAI = [(hip circumference)/((height)1.5)–18)] [32].

### Volume of visceral fat

The volume of visceral fat (VVF) was estimated using the predictive equation proposed by Petribu *et al*., [33] that uses as independent variables the waist-to-height ratio (WHtR) and fasting glucose (FG), as follows:

$$\begin{array}{cc} \mathrm{VVF}= & -130.941+\left(198.673\times \mathrm{WHtR}\right)\\ & +\left(1.185\times \mathrm{FG}\right); \end{array}$$

This equation was developed from a multiple regression analysis by adopting the ultrasonography as a reference standard.

### Blood pressure measurement

Systolic (SBP), diastolic (DBP) and mean blood pressure (MBP) were measured before the initiation of the training program and four days after the RT was finished with an oscillometric device (Microlife 3 AC1-1, Widnau, Switzerland) according to the recommendations of the VI Brazilian Guidelines on Hypertension [25]. The cuff size was adapted to the circumference of the arm of each participant according to the manufacture’s recommendations. SBP and DBP values were used to determine MBP according to the following equation:

$$\mathrm{MBP}=\mathrm{DBP}+\left[\left(\mathrm{SBP}–\mathrm{DBP}\right)/3\right]$$

Heart rate (HR) was measured by a HR monitor (Polar® S810i, Polar Electo Oy, Kempele, Finland). All blood pressure measures were assessed in triplicate (measurements separated by 1 min) with the mean value used for analysis.

### Biochemical parameters

Participants reported to the laboratory between 08:00–10:00 am, after an overnight fast, for blood withdrawal from the antecubital vein. Plasmatic triglycerides, HDL-cholesterol and glucose levels were measured by enzymatic CHOP-POD, homogeneous HDL-cholesterol and Hexokinase methods, respectively. Plasma insulin concentration was measured using a Roche Diagnostics Elecsys 2010 system (Roche Diagnostics, Indianapolis, IN, USA) by the sandwich principle. Glycated hemoglobin (HbA1c) was measured by turbidimetric immunoinhibition on an LX20 analyzer (Beckman Instruments, Brea, CA, USA).

### Resistance training program

**Figure 2:**
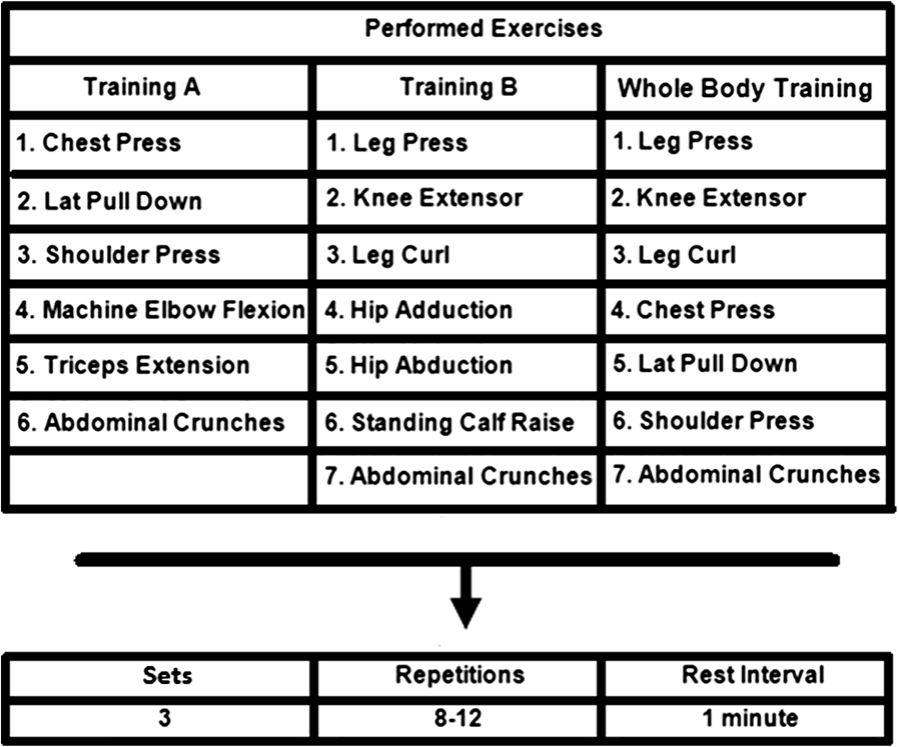
**Division of the RT sessions throughout the study.** For all exercises, 3 sets of 8–12 repetitions maximum were performed with 1-minute rest interval between each set and exercise.

Subjects completed two weeks of familiarization prior to the RT program. In the familiarization weeks individuals:they were advised regarding proper RT technique and completed 3 sessions/week, with one exercise of each main muscle group consisting of 3 sets of 10–12 submaximal repetitions. After the familiarization period subjects initiated The RT program consisting of 3 sessions/week during eight weeks. RT machines were from JOHNSON (Landmark Drive, Cottage Grove, USA). All training sessions were carefully supervised by three experienced professionals (ratio of supervision 1:2 – 1 professor for 2 participants). Participants were required to complete at least 85% of the exercise sessions. No major complications or cardiac events occurred during the study period. Figure 2 shows the exercise order that was strictly followed. The RT was divided into A (Monday) and B (Tuesday) and whole body (Friday) regiments. Abdominal crunches (three sets of 15 repetitions in all sessions) were included. For all listed exercises, three sets with 8–12 RM were performed, with a one-minute rest interval between each set and exercise. Training loads were monitored each session according to the increase in muscle capacity of the participants. The mean duration to complete one repetition was 3–4 s (both concentric and eccentric phases of the movement) and training sessions lasted ≈ 40-50 min. The number of repetitions and the loads used for each exercise session were recorded. The loads were updated when necessary to keep the number of repetitions within the same range of RM and to provide a progressive overload. Additionally, correct breathing patterns were instructed to avoid Valsalva maneuver.

### Statistical analysis

Data are reported as means ± standard deviation (SD). The normal distribution of the data was checked using The Shapiro-Wilk normality test and a homoscedasticity test (Mauchly). The pre and post-intervention variables were compared by using paired Student’s *t*-test and Wilcoxon test for the nonparametric data. In addition, the correlation between delta (post-pre) of muscle strength and thickness with delta (post-pre) of anthropometric and biochemical variables were checked by means of Spearman correlation. The magnitude of differences was verified by the effect size (ES) of Cohen with threshold values of 0.2 (small), 0.6 (moderate), 1.2 (large), and 2.0 (very large) considered. Significance level was set at P < 0.05. Considering the variable muscle strength, the estimated sample size required would be of seven individuals for a Power of 80%. All data were analyzed using the Statistical Package for Social Sciences (SPSS, v.19, Chicago, IL).

## Results

Subjects’ general characteristics are presented in Table 1. There were no statistically significant alterations after eight weeks of RT on the anthropometric and blood pressure variables (P > 0.05). Additionally, ES values were between 0.05 and 0.31, indicating small magnitudes.

**Table 1:** Anthropometric and hemodynamic characteristics of the subjects (n = 14) before and after eight weeks of resistance training

**Variables**	**Pre**	**Post**	**Δ**	**P**	**ES**
Age (y)	33.9 ± 8.6	-	-	-	-
Body mass (kg)	75.5 ± 12.9	76.4 ± 14.1	0.96	0.14	0.07
Height (m)	1.59 ± 0.08	-	-	-	-
BMI (kg·m^2^)	29.6 ± 4.1	29.9 ± 4.2	0.36	0.13	0.09
WC (cm)	88.4 ± 10.1	89.1 ± 8.9	0.77	0.45	0.08
HC (cm)	107.5 ± 8.8	108.0 ± 8.9	0.52	0.45	0.06
NC (cm)	34.9 ± 1.9	35.2 ± 1.7	0.30	0.35	0.15
BAI (%)	27.0 ± 3.9	27.3 ± 3.6	0.21	0.45	0.06
WHpR	0.82 ± 0.07	0.83 ± 0.06	0.01	0.63	0.05
WHtR	0.55 ±0.06	0.56 ± 0.05	0.01	0.45	0.08
VVF (cm^2^)	88.4 ± 22.1	90.0 ± 25.1	1.65	0.61	0.07
SBP (mmHg)	127.9 ± 15.1	123.2 ± 9.6	−4.7	0.19	0.31
DBP (mmHg)	83.7 ± 10.1	81.6 ± 8.7	−2.1	0.15	0.20
MBP (mmHg)	98.4 ± 10.9	95.5 ± 8.7	−2.9	0.11	0.27

BMI, body mass index (kg·m2); WC, waist circumference (cm), HC, hip circumference (cm); NC, neck circumference (cm), BAI, body adiposity index (%); WHpR, waist to hip ratio; WHtR, waist to height ratio; VVF, volume of visceral fat (cm^2^); SBP, systolic blood pressure (mmHg); DBP, diastolic blood pressure (mmHg); MBP, mean blood pressure (mmHg); ES, effect size.

Table 2 presents data from biochemical variables. Again, there were no statistically significant modifications after the RT program on blood glucose (P = 0.38), glycated hemoglobin (P = 0.63), insulin (P = 0.38), triglycerides (P = 0.38), HDL-C (P = 0.69) and triglycerides/HDL ratio (P = 0.74). Magnitudes of the ES were small, between 0.08 and 0.23.

**Table 2:** Biochemical variables of the subjects (n = 14) before and after eight weeks of resistance training

**Variables**	**Pre**	**Post**	**Δ**	**P**	**ES**
Glucose (mg·dL)	89.7 ± 19.4	94.2 ± 19.1	4.5	0.38	0.23
HbAIc (%)	5.29 ± 0.49	5.32 ± 0.54	0.04	0.63	0.08
Insulin (μUI·mL)†	7.32 ± 7.55	11.7 ± 8.5	4.4	0.15	-
Triglycerides (mg·dL)†	95.0 ± 70.7	85.0 ± 41.9	−10.0	0.13	-
HDL (mg·dL)	47.4 ± 19.8	49.8 ± 15.1	2.45	0.69	0.12
TG/HDL ratio†	2.16 ± 1.32	2.25 ± 1.34	0.08	0.74	-

†Values expressed as median; HbAIc, glycated hemoglobin; ES, effect size. HDL, high-density lipoprotein; TG, triglycerides.

Neuromuscular variables are presented in Table 3. There was a significant increase for chest press (P = 0.02), front lat pull-down (P = 0.01), right handgrip and left handgrip (P = 0.02) strength after the RT program. There was an increase in the RF muscle thickness (P = 0.02). Values of ES were of small to moderate magnitude from 0.27 to 0.70. However, there were no statistically significant differences on total muscle mass (P = 0.15) and BB muscle thickness (P = 0.26).

**Table 3:** Neuromuscular variables of the subjects (n = 14) before and after eight weeks of resistance training

**Variables**	**Pre**	**Post**	**Δ**	**P**	**ES**
Chest press (kg)	52.9 ± 9.7	59.8 ± 7.7*	6.8	0.02	0.71
Relative strength	0.71 ± 0.09	0.79 ± 0.15*	0.08	0.03	0.88
Front lat pull-down (kg)	51.5 ± 7.5	57.6 ± 9.2*	6.1	0.01	0.81
Relative strength	0.69 ± 0.11	0.75 ± 0.13*	0.06	0.01	0.56
Right handgrip (kg)	30.1 ± 6.4	31.6 ± 5.5*	1.7	0.02	0.27
Relative strength	0.40 ± 0.09	0.42 ± 0.08	0.02	0.08	0.23
Left handgrip (kg)	28.2 ± 5.6	30.5 ± 4.6*	2.2	0.02	0.40
Relative strength	0.38 ± 0.07	0.40 ± 0.06	0.03	0.07	0.35
Biceps MT (mm)	26.1 ± 5.4	24.8 ± 2.7	−1.3	0.26	0.24
Rectus femoris MT (mm)	42.2 ± 8.5	45.1 ± 7.3*	2.8	0.02	0.33
Total muscle mass (kg)	25.6 ± 3.8	25.8 ± 4.2	0.2	0.15	0.06

*Statistically significant different as compared with Pre, P ≤ 0.05, MT, muscle thickness (mm); ES, effect size.

The correlations among the variables of the study are summarized on Table 4. There were positive correlations of body mass and total muscle mass with relative muscle strength of front lat pull-down (p < 0.05), and inverse correlations of insulin with chest press relative muscle strength (p < 0.05), VVF with relative muscle strength of right handgrip (p < 0.05), triglycerides and TG/HDL ratio with BB muscle thickness (p < 0.05).

**Table 4:** Correlation of delta (post-pre) of muscle strength and thickness with delta (post-pre) of anthropometric and biochemical variables

**Variables**	**Chess press (relative)**	**Front lat pull-down (relative)**	**Right hand grip (relative)**	**Biceps MT (mm)**
Body mass (kg)	NS	0.53*	NS	NS
TMM (kg)	NS	0.53*	NS	NS
VVF (cm^2^)	NS	NS	−0.60*	NS
Insulin (μUI·mL)	−0.60*	NS	NS	NS
TG (mg·dL)	NS	NS	NS	−0.63*
TG/HDL ratio	NS	NS	NS	−0.57*

*Statistically significant (P ≤ 0.05), MT, muscle thickness; TMM, total muscle mass; VVF, volume of visceral fat; TG, Triglycerides; NS, non-significant.

## Discussion

The aim of the present study was to analyze the effects of eight weeks of RT on anthropometric, cardiovascular and biochemical risk factors of MetS, and neuromuscular variables on overweight/obese women. The results of the present study revealed that chronic RT induced an increase of RF muscle thickness and muscle strength, with no effects on anthropometric, cardiovascular and biochemical risk factors of MetS in overweight/obese women. Additionally, there was a correlation between the chronic increase of relative muscle strength with a decrease of triglycerides, TG/HDL ratio, insulin and VVF.

RT is recommended by the American Heart Association [13], the American College of Sports Medicine [14] and the American Diabetes Association [15] as an effective tool to prevent and treat metabolic diseases. These recommendations are based on evidence showing that RT promotes increased muscle mass, glucose transporter (GLUT-4), protein kinase B and glycogen synthase in obese and diabetic individuals [34]. However, fully understanding of the mechanisms responsible for the decrease in blood pressure and dyslipidemia remain to be determined.

On the other hand, results of the present study revealed that despite the increase of RF muscle thickness and muscle strength, no modifications were observed for blood glucose, HDL-C, insulin and glycated hemoglobin. Similarly, meta-analytic data showed that isolated exercise programs without caloric restriction induced limited improvements on cardiovascular risk factors. Shaw *et al*., [35] evaluated 43 studies including 3476 participants and found that exercise without caloric restriction control is associated with a lower decrease of body mass, blood pressure and blood glucose as compared with exercise associated with dietary restriction. Orozco *et al*., [36] compared the effects of isolated diet and diet + aerobic and RT. Results showed that diabetes risk was lower in the combined group. Additionally, individuals submitted to exercise training + diet presented a decrease of blood pressure and anthropometric indexes of obesity, which was not observed for the group of isolated exercise.

Another relevant difference of the present study as compared with other interventions with overweight/obese individuals was that no aerobic exercise was allowed, which may explain the limited results found on risk factors for MetS. This is reinforced by the results from Libardi *et al*., [37], in which overweight individuals completed 16 weeks of aerobic + RT and exhibited increased aerobic capacity and improved lipid profile. In this sense, Ismail *et al*., [38], found that aerobic exercise training induced a decrease in visceral fat, while RT induced no modification on this parameter. Another study from Potteiger *et al*., [39] compared the effects of aerobic exercise at 65–80% of maximum heart rate and RT at 5-10RM with caloric restriction on the cardiovascular risk factor of MetS. The authors reported that aerobic training induced a decrease of MetS z score after six months, while no results were found for RT.

Sigal *et al*., [40] investigated the effects of aerobic, RT and combined (aerobic + RT) training on cardiovascular risk factors in type 2 diabetic individuals. Similar to the results of the present study, there was no alteration on blood pressure, HDL-C, LDL-C and triglycerides after isolated RT. Furthermore, only aerobic and combined training induced significant modifications on body composition. Finally, after 22 weeks of RT associated with dietetic reeducation, there was a decrease of glycated hemoglobin, while combined training induced a superior decrease on glycated hemoglobin compared to only aerobic or RT.

Regarding the neuromuscular variables evaluated in the present study, muscle strength and RF muscle thickness increased after eight weeks of RT. Previous studies revealed that even after brief RT, muscle strength [41] and muscle thickness increase [42,43]. Although muscle thickness determined by ultrasound technique may not differentiate hydration status, muscle, connective tissue and intramuscular fat, this technology is considered a reliable method to measure muscle thickness [44]. A possible explanation for the increase of RF muscle thickness was that more exercises were completed for the lower limb, while only one specific for biceps brachii was included (arm curl). The increase of muscle strength would be expected as initial strength gains (1–8 weeks) due to strength training are primarily neural adaptations [45]. In addition, the increase of muscle strength is of greater importance to the maintenance of functional capacity as compared to other physical variables [46], which may prevent several degenerative process associated with overweight and obesity.

The present study has some limitations that should be considered, such as the limited time of the intervention (only eight weeks), the reduced number of participants and lack of more accurate measures to evaluate body composition. Another limitation was the lack of a control group, although it has been speculated that a control group is not always necessary, particularly considering that the health of the individuals could be compromised as a result of not participating in RT. Additionally, results from a control group would probably reveal no positive effect on anthropometric, biochemical and cardiovascular risk factor of MetS, as has been reported in other studies [23,40,47]. Finally, the non significant variation of the biochemical data, such as glucose and insulin values can be, at least in part, attributed to the lack of dietetic control. Probably, an interdisciplinary approach would induce more positive results on biochemical and anthropometric variables, as previously shown in obese adolescents [48,49].

In summary, eight weeks of RT without caloric restriction had no effects on biochemical, anthropometric and cardiovascular risk factors of MetS in overweight/obese women. However, RT induces important improvement of muscle strength and RF muscle thickness in this population. The increase of muscle strength and lower limb muscle thickness are important outcomes to improve and/or maintain functional capacity during daily living activities, which may prevent chronic degenerative process associated with sedentary lifestyle. It can be hypothesized that longer interventions and the association of RT with aerobic exercise, in addition to the caloric restriction would induce superior results on risk factors of MetS.

## Competing interests

This research received no specific grant from any funding agency in the public, commercial, or not-for-profit sectors. The authors declare no competing interest.

## Authors’ contributions

RAT, AdeOS, CRC, GBP and JP were responsible for concept and design, statistical expertise, data analysis and interpretation, helped write the manuscript. RAT, JN, MB and JP were responsible for data analysis and interpretation and helped write the manuscript. DV, JN, VT, DLdeF, SB and JCdeS were significant manuscript reviewers/revisers and were responsible for data analysis and interpretation. All authors have read and approved the manuscript for publication.
